# Connecting proline metabolism and signaling pathways in plant senescence

**DOI:** 10.3389/fpls.2015.00552

**Published:** 2015-07-22

**Authors:** Lu Zhang, Donald F. Becker

**Affiliations:** Redox Biology Center, Department of Biochemistry, University of Nebraska-Lincoln, Lincoln, NE, USA

**Keywords:** proline, proline dehydrogenase, ^1^Δ-pyrroline-5-carboxylate synthetase, plant senescence, reactive oxygen species

## Abstract

The amino acid proline has a unique biological role in stress adaptation. Proline metabolism is manipulated under stress by multiple and complex regulatory pathways and can profoundly influence cell death and survival in microorganisms, plants, and animals. Though the effects of proline are mediated by diverse signaling pathways, a common theme appears to be the generation of reactive oxygen species (ROS) due to proline oxidation being coupled to the respiratory electron transport chain. Considerable research has been devoted to understand how plants exploit proline metabolism in response to abiotic and biotic stress. Here, we review potential mechanisms by which proline metabolism influences plant senescence, namely in the petal and leaf. Recent studies of petal senescence suggest proline content is manipulated to meet energy demands of senescing cells. In the flower and leaf, proline metabolism may influence ROS signaling pathways that delay senescence progression. Future studies focusing on the mechanisms by which proline metabolic shifts occur during senescence may lead to novel methods to rescue crops under stress and to preserve post-harvest agricultural products.

## Introduction

**FIGURE 1:**
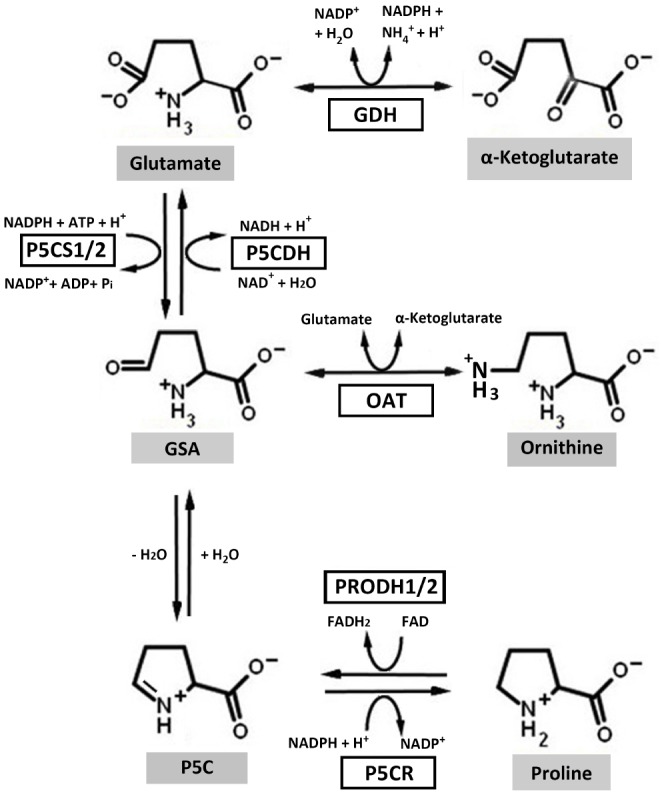
**Proline metabolic pathways in higher plants.** In the biosynthesis pathway, ornithine and glutamate can be converted to glutamate-γ-semialdehyde (GSA) by ornithine-δ-aminotransferase (OAT) and ^1^Δ-pyrroline-5-carboxylate (P5C) synthetase (P5CS), respectively. GSA can then spontaneously cyclize to P5C by losing one molecule of H_2_O. P5C is the substrate for P5C reductase (P5CR), which catalyzes the last step in proline synthesis. In the catabolic pathway, proline dehydrogenase (PRODH) and P5C dehydrogenase (P5CDH) catalyze the oxidation of proline to glutamate. Electrons from reduced flavin (FADH_2_) are transferred to the respiratory electron transport chain to regenerate oxidized flavin (FAD) and complete the PRODH catalytic cycle. Glutamate dehydrogenase (GDH) interconverts glutamate and α-ketoglutarate, which enters the tricarboxylic acid cycle. Higher plants harbor two isoforms of *P5CS* and *PRODH*.

Proline metabolism involves the interconversion of proline and glutamate, a process linked to cellular energetics directly via the respiratory electron transport chain. Proline in higher plants is synthesized from glutamate and ornithine (Fichman et al., 2014). Glutamate-derived proline requires the bifunctional enzyme ^1^Δ-pyrroline-5-carboxylate (P5C) synthetase (P5CS), which catalyzes a two-step reaction requiring ATP and NADPH to generate glutamate-γ-semialdehyde (GSA; Figure 1). GSA spontaneously cyclizes to P5C which is then reduced to proline in a NADPH dependent reaction catalyzed by P5C reductase (P5CR) (*P5CR*; At5g14800; Liang et al., 2013). In higher plant, P5CS, the rate-limiting enzyme of proline biosynthesis, has two isoforms, *P5CS1* (At2g39800) and *P5CS2* (At3g55610). *P5CS1*, localized in the chloroplast, is responsible for stress-induced proline synthesis (Liu et al., 2012a) whereas *P5CS2*, localized in the cytosol, is important for embryo development (Szekely et al., 2008; Funck et al., 2012). Ornithine-derived proline requires ornithine-δ-aminotransferase, which converts ornithine into GSA (Funck et al., 2008; Fichman et al., 2014).

The oxidation of proline to glutamate involves two mitochondrial enzymes, the flavin-dependent proline dehydrogenase (PRODH) and NAD^+^-dependent P5C dehydrogenase (P5CDH) (*P5CDH*; At5g62530; Figure 1; Liang et al., 2013). PRODH catalyzes the rate-determining step of proline catabolism and in plants exists as two isoforms, *PRODH1* (At3g30775) and *PRODH2* (At5g38710). A search of available plant genomes indicates that the two PRODH isoforms are commonly found in higher plants. *PRODH1* is ubiquitously expressed in plant whereas *PRODH2* expression is localized to the vasculature (Funck et al., 2010). Proline oxidation yields approximately 30 ATP due to coupling with the mitochondrial electron transport chain and glutamate entering the tricarboxylic acid cycle as α-ketoglutarate (Verbruggen and Hermans, 2008).

Besides its proteogenic function, proline has roles in energy utilization (Hare and Cress, 1997), reactive oxygen species (ROS) generation (Donald et al., 2001; Szekely et al., 2008; Liu et al., 2012b), programmed cell death (PCD; Donald et al., 2001; Liu et al., 2012b), unfolded protein response (Liang et al., 2014), cell reprogramming and development (Pistollato et al., 2010; Funck et al., 2012; D’Aniello et al., 2015), stress resistance (Strizhov et al., 1997; Krishnan et al., 2008; Szekely et al., 2008; Szabados and Savoure, 2010), and aging (Zarse et al., 2012; Pang and Curran, 2014). In plants, proline metabolism has been proposed to provide stress protection by helping maintain NADPH/NADP^+^ balance, GSH levels, and during pathogen infection, drive the oxidative burst of the hypersensitive response (HR; Miller et al., 2009; Ben Rejeb et al., 2014). Of interest to us are mechanisms by which proline influences the senescence process. Here, we review evidence for proline metabolism having a role in plant petal and leaf senescence.

## Proline in Senescing Petals: Energy Depletion and ROS Accumulation

Senescence is initiated in flowers by natural aging, pollination, and detachment, and culminates in PCD (Rogers, 2012, 2013). Petals, one of the non-productive organs of flowers undergoing senescence (Rogers, 2012), are not photosynthetic (Rogers, 2013). Petals commonly serve as nutrient sinks during development and typically exhibit energy depletion during late stages of senescence (Rogers, 2013). Here, we discuss proline metabolism in the senescing petal of cut flowers.

Studies of flowers have revealed that proline metabolism may have several impacts on petal senescence. A 14-fold increase in proline content was found in petals of cut roses (*Rosa hybrid*) during senescence (Kumar et al., 2009). In rose petals, increased activity of P5CS and PRODH was observed during senescence progression. P5CS activity was generally higher than PRODH at different stages of senescence (Kumar et al., 2009). The elevated P5CS activity indicates that higher proline content was primarily due to biosynthesis from glutamate (Kumar et al., 2009). Glutamate can be generated by coordinated glutamine synthetase (GS) and glutamine oxoglutarate aminotransferase activity at early stages of senescence and, at late-senescence, by glutamate dehydrogenase (GDH; Figure 1; Kumar et al., 2009). The rise in proline content during senescence may be triggered by lower water potential as senescing rose petals were found to have decreased water potential and elevated levels of the stress hormone abscisic acid (ABA; Kumar et al., 2008), a signaling molecule that induces proline biosynthesis during stress (Savoure et al., 1997; Strizhov et al., 1997). It has not yet been fully explored whether increased proline content facilitates petal senescence or is only a consequence of senescence. Interestingly, at complete flower senescence proline levels dropped by 50% suggesting that loss of endogenous proline correlates with the end of senescence and termination of vase life. Exogenous application of proline to petals was thus suggested as a possible approach for extending vase life (Kumar et al., 2009).

Because flowers are heterotrophic, increased PRODH activity likely unleashes proline as a fuel source for ATP production during petal senescence. In cut tulips (*Tulipa gesneriana*), ATP content in petals drops dramatically at day one of flower opening (Azad et al., 2008). Sucrose supplementation, which helps maintain ATP levels, increases vase life indicating the importance of ATP production (Azad et al., 2008). Evidence supporting utilization of proline during senescence was shown by treatment of carnations (*Dianthus caryophyllus*) with aminooxyacetic acid, an inhibitor of ethylene biosynthesis (Yakimova et al., 1997). Aminooxyacetic acid extended vase life, which correlated with a 40% decrease in endogenous proline content of senescing petals compared to untreated flowers (Yakimova et al., 1997). Also, application of the plant hormone salicylic acid (SA) to lisianthus flowers (*Eustoma grandiflorum Mariachi*) doubled the vase life, which correlated with a 75% reduction in proline content (Kazemi et al., 2011). It is possible that the lower proline content observed with SA treatment is due to increased PRODH activity in the petals. In leaves of *Arabidopsis*, exogenous application of SA activates transcription of *PRODH1* (Cecchini et al., 2011). Whether SA also increases PRODH activity in flower petals needs to be confirmed. Application of 5 mM proline extended the vase life of *Rosa hybrida* by 30%, resulting in higher endogenous proline content and PRODH levels (Kumar et al., 2010). Altogether, the results from different studies suggest that proline catabolism and exogenous proline treatment may delay petal senescence by preventing depletion of ATP.

The benefit of proline catabolism during energy-depleted conditions is well known in other organisms and, in worms has been linked to the aging process. For example, in the *Caenorhabditis elegans daf-2* mutant, AAK-2 (AMP-activated kinase, AMPK) upregulates *PRODH*, which is proposed to facilitate lifespan extension by replenishing ATP levels and generating ROS as a signaling molecule to induce antioxidant defenses (Zarse et al., 2012). The AMPK homolog in plants is SnRK1 (sucrose-non-fermenting-1-related protein kinase-1), which regulates members of the S1 basic leucine zipper (bZIP) transcription family, such as *bZIP1*, *bZIP11*, and *bZIP53*, under low sugar or energy conditions (Polge and Thomas, 2007; Tome et al., 2014). *bZIP11* is expressed more abundantly in petals (Hummel et al., 2009) relative to *bZIP1* and *dZIP53* (Llorca et al., 2014). Overexpression of *bZIP11* resulted in decreased proline content consistent with upregulation of proline catabolism (Hanson et al., 2008). In *Arabidopsis*, expression of *PRODH2* in the vascular tissue and abscission zone of petals is regulated by bZIP11 (Hanson et al., 2008) whereas *PRODH1* expression is induced by bZIP1 and bZIP53 (Dietrich et al., 2011). Thus, in response to low sucrose, expression levels of *dZIP1*, *bZIP11*, and *bZIP53* increase, resulting in higher expression of *PRODH1* and *PRODH2* thereby increasing proline catabolic flux (Funck et al., 2010; Llorca et al., 2014).

Is regulation of PRODH by bZIPs relevant to senescence? A role for SnRK1 and bZIPs in senescence is supported by different studies. For example, disruption of SnRK1 was shown to accelerate senescence progression in moss (*Physcomitrella patens*, Polge and Thomas, 2007). The expression of *bZIP1* and *bZIP53* in *Arabidopsis* are upregulated during dark-induced leaf senescence (Dietrich et al., 2011). *PRODH1*, regulated by bZIP1 and bZIP53, is the dominant isoform in flowers under most conditions (Funck et al., 2010). Therefore, bZIP1, bZIP53, and PRODH1, may have important roles in the mechanism by which SnRK1 influences the senescence of cut flowers. More direct evidence is needed to establish whether PRODH1 and PRODH2 have a critical role in senescence delay via the SnRK1 signaling pathway.

Besides energy depletion, another important factor of senescence is the accumulation of oxidative damage in aging tissue. Studies have shown that ROS accumulation plays a vital role in flower senescence as reviewed by Rogers (2012). ROS appears to facilitate the aging process and is necessary for petal senescence. H_2_O_2_ was found to buildup in daylilies (*Hemerocallis*) during senescence progression (Chakrabarty et al., 2009). In *Tulipa gesneriana*, rising H_2_O_2_ levels followed the appearance of senescence markers (protease activity and cytochrome c) at the end of flower opening (Azad et al., 2008). To alleviate the oxidative burden in snapdragon (*Antirrhinum majus*) petals, ascorbic acid was applied as an exogenous antioxidant resulting in a 20% longer vase life relative to untreated flowers (Abdulrahman et al., 2012).

Proline is known to protect against oxidative stress in many organisms including fungi (Chen and Dickman, 2005; Chen et al., 2006), bacteria (Zhang et al., 2015), plants (Szabados and Savoure, 2010; Sorkheh et al., 2012), and to animals (Natarajan et al., 2012; Zarse et al., 2012). It is still controversial, however, whether protection is due to proline directly scavenging ROS. Proline is expected to efficiently react with ^•^OH (Signorelli et al., 2014) and was suggested to scavenge ^1^O_2_ (Alia et al., 2001). Another study, however, reported that proline does not quench ^1^O_2_ in plants (Signorelli et al., 2013) and recently, proline was found not to directly scavenge H_2_O_2_ (Zhang et al., 2015). In bacteria and mammalian cells, PRODH is necessary for proline-mediated adaptation to oxidative stress (Natarajan et al., 2012; Zhang et al., 2015). Proline catabolism generates H_2_O_2_ as a by-product thereby activating antioxidant signaling pathways. Proline oxidation induces the OxyR regulon of *Escherichia coli* (Zhang et al., 2015), Akt pathway in human cells (Natarajan et al., 2012), and the MAPK pathway (Okuyama et al., 2010) in the *C. elegans daf-2* mutant (Zarse et al., 2012). Thus, although the signaling pathways vary, H_2_O_2_ production by proline catabolism seems to be a conserved mechanism by which proline influences antioxidant defenses.

Could proline metabolism influence ROS accumulation during senescence? Interestingly, relative to untreated roses, proline treatment sustained Mn-dependent superoxide dismutase (Mn-SOD) activity, the dominant SOD in petals, resulting in twofold lower levels of superoxide anion radicals at each stage of petal senescence (Kumar et al., 2010). In plant, MAPK cascades respond to ROS and regulate antioxidant signaling pathways (Pitzschke et al., 2009; Sinha et al., 2011). MAPK cascades consist of three kinases: MAPK kinase kinases (MEKKs), MAPK kinases (MKKs) and MAPKs (MPKs; Sinha et al., 2011). H_2_O_2_ activates ROS scavenging enzymes by initiating a phosphorylation cascade involving MKK4/5 and MPK3/6 (Kovtun et al., 2000). MKK4 and MKK5 are known to be necessary for flower organ abscission in *Arabidopsis* (Cho et al., 2008). Tandem RNAi knockdown of both *MKK4* and *MKK5* showed a defect in petal abscission, although individual RNAi knockdown of either gene had a normal phenotype (Cho et al., 2008). MPK3 and MPK6 were also required for petal abscission (Cho et al., 2008). Future studies are needed to understand whether ROS triggers the phosphorylation of MKK4/5 and MPK3/6 during petal senescence thereby promoting petal abscission.

Little is known about the relationship between PRODH and the MAPK pathway in plants, however, hypoosmotic stress was observed to induce a similar pattern of *MPK20* and *PRODH* expression in *Arabidopsis*, suggesting a link between PRODH and MPK20 (Moustafa et al., 2008). A recent genomic study showed *MPK20* was highly induced by H_2_O_2_ stress in cotton (*Gossypium raimondii*, Zhang et al., 2014). Is it plausible that ROS generated by proline metabolism induces *MPK20* during petal senescence? Stronger evidence is available for linking proline biosynthesis with MAPK signaling, namely MKK4. Overexpression of *ZmMKK4*, a gene from *Zea mays*, in tobacco or *Arabidopsis* resulted in elevated P5CS2 activity (Kong et al., 2011a), leading to increased proline content and tolerance to hyperosmotic stress (Kong et al., 2011a,b). In line with the ROS sensing role of the MAPK signaling pathway, overexpression of *ZmMKK4* resulted in higher peroxidase activity and lower ROS levels (Kong et al., 2011a,b). Whether MKK4 also upregulates *P5CS* during flower senescence needs to be determined.

## Proline in Leaf Senescence: Upregulation of Proline Catabolism and Hormone-Induced Pathways

Leaf senescence, a developmentally regulated PCD, is age-dependent and induced by environmental signals (drought, detachment or darkness; Smart, 1994; van Doorn and Woltering, 2004). The role of proline metabolism in leaf senescence shares some similarities with petals but also has unique features (Price et al., 2008). Proline levels increase proportionally with leaf age in excised leaf segments and are an indicator of leaf senescence (Wang et al., 1982; Mondal et al., 1985). Unlike flowers, leaf generates energy via photosynthesis which is maintained during late stages of senescence (Smart, 1994; Kotakis et al., 2014). Thus, proline is not needed as an energy source during leaf senescence.

What are the relevant mechanisms of proline metabolism in leaf senescence? One observation is that during natural leaf aging proline catabolism appears to be upregulated via increased expression of *PRODH2* and *P5CDH*. In *Arabidopsis thaliana* and *Brassica napus* (rapeseed), *PRODH2* expression was strongly induced in the course of natural leaf aging (Funck et al., 2010; Faes et al., 2015), whereas *PRODH1* expression was moderately upregulated (Funck et al., 2010). *P5CDH* expression was also reported to increase in older leaves of *Arabidopsis* (Deuschle et al., 2004). Why proline catabolism would be upregulated during natural leaf senescence remains largely unresolved since there does not seem to be a significant energy deficit as mentioned above. Proline degradation, however, may assist nitrogen recycling in the phloem from old leaves to sink organs (Faes et al., 2015) which would be consistent with the stronger expression of *PRODH2* in vascular tissues at senescence. Cytosolic *GS1* and *GDH* are also induced during leaf senescence, apparently to facilitate nitrogen recycling (Masclaux-Daubresse et al., 2005). In fact, proline treatment of *Arabidopsis* was observed to induce expression of *GS1* and *GDH* (Masclaux-Daubresse et al., 2005), indicating that proline catabolism provides glutamate as a substrate for GS1 and GDH. Thus, proline may be needed for nitrogen cycling during leaf senescence.

The possibility of PRODH1 being involved in plant hormone induced senescence is inferred from a recent study showing proline metabolism is regulated by phosphatidylinositol-3,4,5-triphosphate dependent kinase (PI3K; Leprince et al., 2015). Under salt stress, inhibition of PI3K by LY294002 resulted in lower *P5CS1* and higher *PRODH1* expression and, decreased proline content in *Arabidopsis* (Leprince et al., 2015). *PRODH1* expression was also higher in a *pi3k*-hemizygous *Arabidopsis* mutant (Leprince et al., 2015). Thus, PI3K appears to repress *PRODH1* expression. The mechanism by which plant hormones such as methyl jasmonic-acid (JA; Hung et al., 2006) and ABA (Hung and Kao, 2005) induce senescence, involves PI3K signaling and H_2_O_2_. Inhibiting PI3K activity with LY294002 aborted H_2_O_2_ production and delayed JA induced-senescence in rice leaves (Hung et al., 2006). Phosphatidylinositol-3-phosphate (PI3P), the product of PI3K, has also been shown to be necessary for ABA-induced H_2_O_2_ production and senescence (Hung and Kao, 2005). Thus, PI3K and PI3P promote plant hormone-induced senescence via H_2_O_2_ production. Repression of *PRODH1* by PI3K could potentially increase oxidative stress burden as discussed above due to loss of proline-mediated ROS signaling and decreased antioxidant defense.

Additional evidence for proline metabolism being involved in plant hormone-induced senescence is from studies of lipid degradation. Lipid degradation is generally upregulated during leaf senescence due to loss of cellular membrane integrity (Lim et al., 2007). Phospholipase D (PLD) catalyzes the production of membrane-bound phosphatidic acid (PA) from phospholipid (Kolesnikov et al., 2012). PLD activity has been shown to gradually increase at senescence in castor oil plant (*Ricinus communis*) leaves (Ryu and Wang, 1995). Plants harbor three isoforms of PLDs: PLDα, PLDβ and PLDδ (Kolesnikov et al., 2012). Antisense suppression of *PLDα*, the most abundant PLD, slowed down ABA- and ethylene-induced senescence of detached *Arabidopsis* leaves, while there was no effect on natural aging leaf senescence (Fan et al., 1997). The suppression of *PLD*δ, which is upregulated during senescence, also delays ABA-promoted senescence in *Arabidopsis* (Jia et al., 2013). PLD appears to be a negative regulator of *P5CS1* as inhibition of PA signaling by 1-butanol was observed to increase *P5CS1* expression in *Arabidopsis* (Thiery et al., 2004) and *Thellungiella halophila* (Ghars et al., 2012). Because senescence-specific degradation of Calvin cycle enzymes has been shown to lower NADP^+^ levels in chloroplasts (Zhang et al., 2012) increased *P5CS1* expression may help maintain NADPH/NADP^+^ balance (Liang et al., 2013). Whether regulation of *P5CS1* by PA signaling is important during hormone-induced leaf senescence needs to be determined.

## Summary and Future Direction

The various roles of proline metabolism in energetics, ROS signaling, and cellular processes continue to unfold (Ben Rejeb et al., 2014). Proline metabolic ROS production appears to be a general phenomenon in diverse organisms potentially impacting cellular processes such as aging and plant senescence. An excellent example of PRODH mediated ROS production in plants is from studies of *Pseudomonas syringae* pathogen induced HR (Cecchini et al., 2011). In the late stages of HR, *PRODH* and *P5CDH* expression becomes uncoupled, thereby enabling proline/P5C cycling via PRODH and P5CR, leading to an oxidative burst (Cecchini et al., 2011; Monteoliva et al., 2014).

**FIGURE 2:**
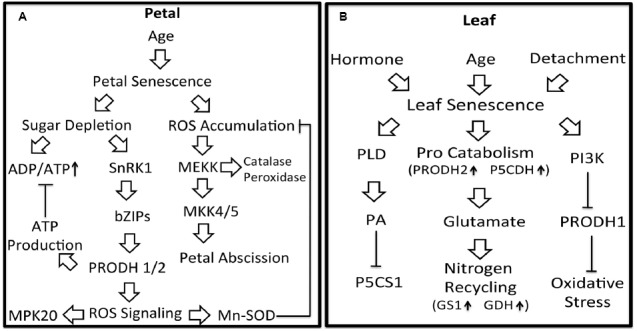
**Potential linkages between proline metabolism and signaling pathways in petal and leaf senescence. Petal senescence (A)**: Aging induced petal senescence results in ROS accumulation and sugar depletion. SnRK1, activated in response to depleted sugar, is proposed to induce *PRODH1/2* expression via bZIP1, bZIP11, and bZIP53. Upregulation of *PRODH1/2* expression would be predicted to generate ATP thereby attenuating increases in ADP/ATP. PRODH activity is also expected to generate ROS as a by-product, possibly leading to activation of MPK20 and increased Mn-SOD activity. Enhanced Mn-SOD activity would help diminish accumulated ROS and oxidative damage during petal senescence. Activation of MAPK pathways by ROS would induce expression of antioxidant enzymes and petal abscission. Leaf senescence **(B)**: Leaf senescence can be induced by plant hormones, age and detachment. During age-related senescence, the expression of *PRODH2* and *P5CDH* are induced, suggesting a higher flux of proline catabolism and more glutamate available for nitrogen recycling. In response to H_2_O_2_ during hormone-induced senescence, PI3K may down-regulate *PRODH1* resulting in less ROS signaling and adaptation to oxidative stress. Also during hormone-induced senescence, phospholipase D (PLD) and its product phosphatidic acid (PA) inhibit *P5CS1* expression.

The molecular pathways by which proline metabolism is regulated during plant senescence are summarized in Figure 2. The ability of proline to delay senescence in petal and leaf tissues is likely due to protection against oxidative stress that occurs in the aging tissue. In leaf, proline may help redistribute nitrogen to younger tissue whereas in flowers, proline helps counter energy shortages. Further insights into how proline metabolism impacts petal and leaf senescence will require additional studies that connect proline with plant senescence signaling pathways. Exploration of the linkages between proline metabolism and important pathways of plant senescence such as MAPK signaling, the SnRK1-bZIP pathway and PI3K signaling will be valuable targets for future study. Better understanding of proline metabolism in senescing leaves may uncover novel strategies for preserving post-harvest flowers and delaying stress-induced leaf senescence.

### Conflict of Interest Statement

The authors declare that the research was conducted in the absence of any commercial or financial relationships that could be construed as a potential conflict of interest.
